# SCIg vs. IVIg: let’s give patients the choice!

**DOI:** 10.1186/1710-1492-10-S2-A13

**Published:** 2014-12-18

**Authors:** Kathryn K Samaan, Marie-Claude RN Levasseur, Hélène Decaluwe, Claire St-Cyr, Hugo Chapdelaine, Anne Des Roches, Elie Haddad

**Affiliations:** 1 Department of Pediatrics, University of Montreal, Centre Hospitalier Universitaire Sainte-Justine, Montreal, Canada; 2 Department of Allergy, University of Montreal, Centre Hospitalier Universitaire de Montreal, Montreal, Canada; 3 Department of Microbiology and Immunology, University of Montreal, Centre Hospitalier Universitaire Sainte-Justine, Montreal, Canada

## Purpose

Criteria governing the choice between intravenous (IV) and subcutaneous (SC) routes for immunoglobulin (Ig) substitution are not well defined. We assessed the consequences of giving the choice to the patient.

## Methods

We retrospectively analyzed 143 patients with primary immunodeficiency, followed in a single center, which were offered the choice of IVIg or SCIg. We analyzed the route more frequently chosen, and the consequences on compliance. In a first cohort (n = 51, average follow up 52 months), patients already on IVIg were offered the choice to stay on IVIg or to switch to SCIg (switch cohort). In a second cohort (n = 92, average follow up 11 months), newly diagnosed patients were offered the choice between IVIg and SCIg before the first injection (new cohort).

## Results

In the switch cohort, 50/51 patients chose to switch to SCIg. Of these, 90% remained on SCIg. In the new cohort, 44/92 patients chose SCIg, of which 95% remained on SCIg. Among the 48 patients who chose IVIg, 73% switched to SCIg. Compliance issues were observed in only 10 patients.

## Conclusion

Giving patients the choice of treatment modality is a safe strategy in terms of compliance. Home-based SCIg is much more frequently chosen than hospital-based IVIg. Given the equal efficacy and safety between hospital-based IVIg and home-based SCIg, we believe that all patients should be given the choice regardless of physician’s belief of “idealness” of the candidate.

